# Reply to "Thirty Years a Hospital Superintendent."

**Published:** 1912-01-20

**Authors:** P. Michelli

**Affiliations:** Secretary Dreadnought Hospital. Greenwich S.E.


### REPLY TO "THIRTY YEARS A HOSPITAL SUPERINTENDENT."

Sir,—I should like to reply to the criticisms which have been made by "Hospital Superintendent" upon my letter in your last issue.

The object for which I appeal may be exceptionally popular, but this fortunate position is not reflected in the amount of legacies received, which is much smaller than most institutions with an equal expenditure; nor have I the advantage of being able to make a special claim upon the locality in which the hospital is situated, or of getting into personal relations with our contributors. I believe, therefore, that the position of this hospital as regards income is much the same as that of other London hospitals.

I assumed when writing my letter that you wished to know my opinion of the position of the hospitals under the Bill as it now stands; and, speaking from this point of view alone, I do not consider that the treatment of insured persons as in-patients constitutes any abuse.

Whatever Mr. Lloyd George has said, or on whatever German Acts his Bill may have been framed, it does not give to the insured person adequate relief in case of serious illness or surgical operation. In the future, as in the past, the breadwinner will have to look to the hospital for treatment under such conditions, and his only advantage under the Bill is that his dependants will get a weekly sum which may keep body and soul together.

I agree with your correspondent that voluntary hospitals are intended to provide for breadwinners who, when well, are able to maintain their dependants, but who cannot defray the cost of medical or hospital treatment. The Act does not give the insured any power to meet the cost of serious illness, and they appear to me therefore to remain suitable cases for hospital treatment.

By increasing the amount of money at the disposal of the Insurance Committees or the Commissioners it would, of course, be perfectly possible to pay the hospitals an adequate *pro rata* sum for their treatment of the insured; but as the Act now stands the financial provision does not permit of any adequate payment, and therefore I think our position would be stronger if we ask for a voluntary subscription from the approved society rather than an inadequate *pro rata* sum carrying with it all the disadvantages attaching to a compulsory payment.

The Act does not appear to me likely to reduce seriously the ability of those who now contribute to hospitals to continue their support, and I take an optimistic view because I believe that the public can be convinced that hospitals will have as much work and require as much assistance after the Act comes into force as before.

There seems to be a good deal of confusion in the mind of your correspondent as to the intention of my letter; he speaks freely of an amending Act. I write only on the Act as it stands; whatever the intentions of the Act or the promises of its originator, I deal only with its performances.

You would not wish me, I think, to deal here with the chances of our getting an amending Act, or what form it should take; and I will close this letter with an earnest appeal to my fellow secretaries not to encourage the withdrawal of subscriptions as a lever to obtain amendments which may not be secured, and which by greatly increasing the financial burden of the Act may make our position as voluntary institutions no longer tenable.—Yours faithfully,

Greenwich, S.E. January 15. P. Michelli, Secretary Dreadnought Hospital.

